# Network structure shapes the impact of diversity in collective learning

**DOI:** 10.1038/s41598-024-52837-3

**Published:** 2024-01-30

**Authors:** Fabian Baumann, Agnieszka Czaplicka, Iyad Rahwan

**Affiliations:** Center for Humans and Machines, Max Planck Institute for Human Development, Lentzeallee 94, Berlin, 14195 Germany

**Keywords:** Computational science, Cultural evolution

## Abstract

It is widely believed that diversity arising from different skills enhances the performance of teams, and in particular, their ability to learn and innovate. However, diversity has also been associated with negative effects on the communication and coordination within collectives. Yet, despite the importance of diversity as a concept, we still lack a mechanistic understanding of how its impact is shaped by the underlying social network. To fill this gap, we model skill diversity within a simple model of collective learning and show that its effect on collective performance differs depending on the complexity of the task and the network density. In particular, we find that diversity consistently impairs performance in simple tasks. In contrast, in complex tasks, link density modifies the effect of diversity: while homogeneous populations outperform diverse ones in sparse networks, the opposite is true in dense networks, where diversity boosts collective performance. Our findings also provide insight on how to forge teams in an increasingly interconnected world: the more we are connected, the more we can benefit from diversity to solve complex problems.

## Introduction

Nowadays, humans communicate more than ever before^1^. Often this communication takes place in order for humans to learn from each other or solve a problem together. With the aim of optimising such collective problem solving, previous studies have focused on the effects of network structure on the overall performance of a population. It has been found that groups do not necessarily benefit from close-knit networks where information can be shared freely and disseminated quickly. Instead, collective problem solving can only benefit when the characteristics of the network, in particular its link density, are adapted to the characteristics of the individual problem solvers as well as to the structure of the problem^2–11^. Specifically, it was shown that collective performance can be boosted by reducing the network’s connectivity, especially when the task to be solved is complex^2,5,6^. Apart from the growing interconnectedness of social networks, the rise in global connectivity also results in individuals from diverse backgrounds and cultures engaging in interactions and collaborations to jointly solve problems^12^. It is therefore crucial to examine not only how network structure affects collective performance, but also how individual differences within networked groups or societies—often referred to as diversity—shape the success of a group. Empirical studies have found contradictory, i.e., positive *and* negative, effects of diversity on the performance of groups and larger organisations^11,13–25^. For example, greater occupational diversity predicts higher productivity of cities^14,26^, and within organizations, the teams with the greatest diversity of educational backgrounds have been found to be the most innovative^13^. Furthermore, it was shown that diverse teams are less prone to detrimental groupthink^24^. However, there is also evidence that diversity can negatively impact collective performance. Specifically, diversity may impair the ability of groups to communicate and coordinate^18–21^, be associated with reduced trust between individuals^14,22^, and can be a source for social tension and conflict^15,23^. Overall, there is limited mechanistic understanding of how network structure *and* diversity together affect the collective performance of a group, as previous modelling attempts have mainly focused on homogeneous populations of identical individuals^2,3,27^.

**Figure 1:**
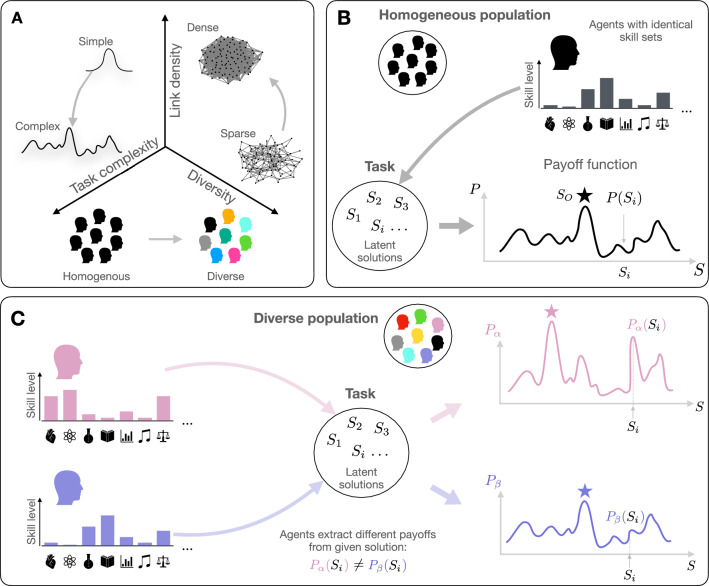
Illustration of the model. Panel (**A**) depicts the features of the model that are varied: (i) task complexity, (ii) link density of the underlying network, and (iii) the composition of the population in terms of skill diversity. In panels (**B**,**C**), we schematically depict how agents’ skills map onto payoff functions *P*. In a homogeneous population, all agents have the same set of skills (panel **B**). This results in a *single* payoff function, which maps a latent solution $S_i$ to payoff value $P(S_i)$ an agent is able to extract from it. In homogeneous populations, all agents can extract the same payoff from a given solution $S_i$, and hence there is one optimal solution $S_O$ (star symbol) for all agents. In panel (**C**), we show the case of a diverse population, where agents generally have different distributions of skills. For brevity, we depict two agents with different skill sets and their corresponding distinct payoff functions $P_\alpha$ and $P_\beta$. As depicted two agents in diverse populations will generally not extract the same amount of payoff from a particular solution $S_i$. Accordingly, also their optimal solutions differ (shown as star symbols).

In this paper, we address this gap by expanding upon an established model of collective problem-solving^2,3^. This extension involves incorporating diverse agents, allowing us to systematically examine the relationship between diversity and network structure, and how this interaction influences group performance in tasks of different complexity. The model is based on the paradigm of social learning, which has previously also been used in empirical studies of collective problem-solving including experiments on the evolution of culture^5,6^, and more narrow cases where groups solved specific real world tasks^27,28^. The basic idea of social learning is that individuals do not solve a problem alone. Instead, they exchange information with each other, a process that can dramatically increase the quality and speed of problem solving. However, it is unclear how network structure shapes the impact of diversity on collective performance. Therefore, we investigate the dynamics of collective learning along three dimensions: (1) task complexity (simple, complex), (2) network structure (sparse, dense), and (3) group composition with regard to skill diversity (homogeneous, increasingly diverse), as depicted in Fig. 1A. Consistent with empirical studies^13–17^, we find strong effects of diversity on collective performance, and we demonstrate that those effects are mixed, i.e., groups may benefit but also suffer from skill diversity. In the model, positive and negative effects of diversity arise as a result of varying the task complexity and the network structure. Diversity generally reduces the performance of groups in simple tasks. In the case of complex tasks, the link density of the network modifies the effect of diversity: the more we are connected, the more we can benefit from diversity to solve complex problems.

## Model

### Setup and dynamics

Following previous work^2,3^, we model collective learning as a process where a population of *N* interacting agents jointly search a space of solutions. In this mathematical metaphor one assumes that solutions to a problem differ in their quality and the collective dynamics is tuned to discover high-quality solutions and spread them throughout the population. We will use the word *payoff* to denote the quality measure that is used to compare two solutions: a solution $S_i$ has a higher quality than a solution $S_j$ if it has a higher payoff as quantified by a function *P*, i.e., $P(S_i)>P(S_j)$.

The interactions between agents are modelled using (social) networks, as both empirical and theoretical studies have shown that the structure of the communication network strongly influences collective problem solving^2–5,27,29,30^. Specifically, we consider random networks as a simple way to control the link density covering a range from very sparse to denser networks. Each agent in the population is associated to a node in the network, which thus defines an agent’s social environment, i.e. the subset of other agents in the population with which the agent can exchange information. For simplicity, we assume that two connected agents can learn equally well from each other and therefore consider undirected networks.

The collective learning dynamics evolves as follows. In each discrete simulation time step *t*, agent *i* aims to improve their solution in a two-step procedure that strikes a balance between *exploitation* and *exploration*, which typically leads to an iterative improvement of group performance^2–4,31–33^. First, the agent engages in exploitation, i.e., they attempt to learn (or copy) a solution from a neighbour in the social network. As in previous studies (e.g. Refs.^2–4^), we implement a “best-member” learning strategy in which the agent first searches for the best solution among its neighbours and compares it with its current solution. If the neighbour’s solution has a higher payoff than the agent’s current solution, it is adopted. Otherwise, the agent *i* switches to exploration, where they aim to innovate a new solution; a process which is modeled as a local search in the solution space (see below). As in the exploitation step, the innovated solution is only adopted if it is associated to a higher payoff than the agent’s current solution^2,3,34,35^. In line with previous works^2–4^, we quantify the collective performance of the population at time *t* as the average of agents’ current payoffs, $\langle P\rangle (t) = N^{-1}\sum _{i=1}^{N} P_i(t)$, where $P_{i}$ corresponds to the contribution of agent *i*.

### Task complexity

Real-world tasks come in different complexities and it has been shown both in computational as well as empirical studies that task complexity strongly impacts collective performance^2,3,28^. Here we use the NK model^35^ that has previously been applied in theoretical studies of social learning^2,3^. The strength of the NK model is that it allows us to flexibly tune the difficulty of a task—from simple to difficult—by changing the properties of the solution space as illustrated in Fig. 1A. In particular, a simple problem corresponds to a smooth payoff function with a single peak which is associated to the optimal solution $S_O$, i.e., the solution with the highest payoff^3,35^. Complex tasks, by contrast, are characterized by a rugged payoff landscape. While such rugged landscapes also have an optimal solution $S_O$, they are characterized by additional peaks that are associated to locally optimal but globally suboptimal solutions. What makes complex tasks harder to solve—both individually and collectively as a group—is the fact that agents often converge to such suboptimal solutions^2,3,28^.

The NK model is fully defined by two parameters: $N_{\rm NK}$ and $K_{\rm NK}$. Each solution *S* is given by a vector of $N_{\rm NK}$ binary elements $b_i$, or bits (0 or 1), and therefore the total number of possible solutions is $2^{N_{\rm NK}}$. The payoff of each solution *S* is defined as the average payoff contributions of each element $b_i$, which is a uniformly distributed random number between 0 and 1 and is computed using the function *f*. Crucially, for $K_{\rm NK}=0$, the payoff contribution *f* of a single element, $b_i$, only depends on the state of that element (0 or 1), i.e. we have $f(b_i)$, yielding $P=N^{-1}\sum _i f(b_i)$. By contrast, for $K_{\rm NK}>0$, the payoff contribution of element $b_i$ is interlinked to the states of $K_{\rm NK}$ elements of the vector, i.e., we have $f(b_i\vert b_i, b_{i+1}, \dots ,b_{K_{\rm NK}})$ yielding $P=N^{-1}\sum _i f(b_i\vert b_i, b_{i+1}, \dots ,b_{K_{\rm NK}})$, where the $K_{\rm NK}$ elements are determined randomly. Previously, it has been observed that for intermediate values of $K_{\rm NK}$ the distribution of payoffs tends towards a normal distribution, such that most solutions have similar payoffs, which makes it hard to distinguish the collective performance in different conditions^3^. We therefore first normalize the payoff function *P* by its maximum value $P_{\rm max}$^2,3,36^ and take the normalized payoff function to the power of 8, such that there are only very few high-quality solutions^2,3^. We distinguish between two scenarios: simple and complex tasks for which we set the parameters of the NK model to $(N_{\rm NK}, K_{\rm NK}) = (15,0)$ and $(N_{\rm NK}, K_{\rm NK})= (15, 7)$, respectively.

In line with previous research^2,3^, we describe agents’ exploration (or innovation) as a constrained search process within the solution space. This means that an agent can only create or uncover new solutions that are close to their current solution $S_i$. In the context of the NK model, or more broadly, in the realm of binary vectors, the nearest solutions to a given solution $S_i$ are those that differ in just one binary element. Consequently, when an agent seeks to innovate and generate a new solution, they do so by altering the state of a single element within $S_i$. To account for the fact that new innovations often occur by chance, the choice of which element to change is typically determined randomly^2,3^.

### Homogeneous and diverse populations

Previously, theoretical works on social learning mainly considered homogeneous populations, where all agents are assumed to be identical^2,3,27^. This uniformity implies that every agent possesses the same distribution of skills, which ultimately leads to a single payoff function *P*, see Fig. 1B. Thus, each agents derives the same payoff value, $P(S_i)$, from a particular solution $S_i$.

In diverse populations the situation is different and agents vary in terms of their skills, as exemplified in panel C of Fig. 1 which shows two agents with differing skills. An important assumption of our model is that the skills of agents and the task combined give rise to a payoff function that is used by agents to navigate the solution space (Fig. 1). This means that—in diverse populations—agents are associated to different payoff functions $P_\alpha$, and therefore, generally do not extract the same amount of payoff from a particular solution $S_i$, $P_\alpha (S_i) \ne P_\beta (S_i)$. Consequently, in the case of diverse populations there is not a single optimal solution for all agents. Instead, different skill sets naturally lead to different solutions spaces and therefore different optimal solutions (star symbols), see Fig. 1C. As an illustration, let us consider a multi-disciplinary team where members tend to concentrate on solutions that align with their skills. For instance, an individual with strong analytical abilities is likely to derive greater benefits (high payoff) from a solution related to a mathematical problem. On the other hand, a team member who excels in linguistics should direct their attention on a solution related to language, aiming to maximize their contribution to the overall team performance.

**Figure 2:**
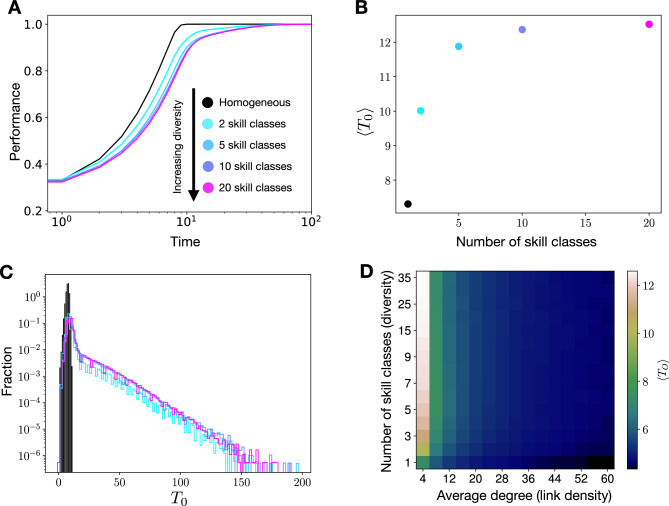
Collective performance in simple tasks. Panels (**A**–**C**) show the average payoffs over time (**A**), average times $\langle T_O\rangle$ to discover the globally optimal solutions (**B**), and the normalized histograms of $T_O$ (**C**) for homogeneous and (increasingly) diverse populations on sparse networks with an average degree of $\langle k\rangle =4$. Panel (**D**) depicts $\langle T_O\rangle$ (color coded) as a function of the average degree of the underlying network and the level of diversity. The reported results are averages over 2500 realizations.

### Simulations

Unless otherwise specified, we consider populations of $N=1000$ agents. At the beginning of each simulation, we randomly assign each agent to a particular network node of a given network. Each agent initially has a solution $S_i$ that is randomly chosen from the total set of $2^{N_{\rm NK}}$ solutions.

In diverse populations, we assume that populations can be divided into equally sized classes of agents with identical skills, i.e. identical payoff functions $P_\alpha$. Each payoff function $P_\alpha$ is generated by the NK model using the same parameters. By increasing the number of skill classes, we therefore increase the degree of diversity in a population. For example, in the case of 1000 agents and assuming two skill classes ($\alpha$ and $\beta$) the population will consists of two groups of agents, each of size 500, and each agent is randomly assigned to one of the two payoff functions, $P_\alpha$ or $P_\beta$.

In our simulations, we consider a synchronous updating of the system. This means that in each discrete time step *t* each agents performs one exploitation and one exploitation step based on the current distribution of solutions. Subsequently, the states of all agents are updated.

## Results

### Simple tasks

Figure 2 shows results obtained for simple tasks. In panels A–C, we consider populations coupled via sparse random networks with an average degree of $\langle k\rangle =4$, and colors encode different levels of diversity, where diversity increases with the number of skill classes (see legend of panel A). In panel D, we vary both the level of diversity and the average degree of the underlying network.

Specifically, Fig. 2A depicts the collective performance quantified as the average payoff $\langle P\rangle$ over time. We find that all populations, both homogeneous (black) and diverse ones (color coded), eventually converge to the solutions $S_O$ with maximum payoff, $P(S_O)=1$. However, the time that populations need to reach $S_O$ depends on the level of diversity. To quantify this effect further we first define $T_O$ as the time that an agent needs to find $S_O$. In Fig. 2B, we depict the mean values of $\langle T_O\rangle$ as a function of diversity (number of skill classes). Clearly, $\langle T_O\rangle$ is smallest in homogeneous populations, increases with diversity, and saturates for high levels of diversity.

In Fig. 2C, we show the full distributions of $T_O$ for agents in homogeneous and diverse populations. For homogeneous populations the distribution of $T_0$ is narrowly centered around a small value (black). Instead, for diverse populations the distributions are much broader and characterized by a pronounced positive skew (color coded). Already for low levels of diversity, i.e. 2 skill classes (cyan line), the distribution of $T_0$ strongly deviates from a narrow distribution that is associated to the homogeneous case.

Finally, Fig. 2D shows $\langle T_O\rangle$ as a function of diversity and the link density of the network, which is tuned by the value of the average degree $\langle k\rangle$. While for increasing $\langle k\rangle$, the effect of diversity becomes less pronounced the qualitative behavior is the same: as populations become more diverse, the average time $\langle T_O\rangle$ to reach optimal solutions increases. We also find that to solve simple problems collectively dense networks are generally beneficial as agents converge faster to optimal solutions in networks with larger values of $\langle k \rangle$. This trend can be observed for all levels of diversity, varying along the vertical axis in panel D of Fig. 2.

Taken together, the results reported in Fig. 2 suggest that in simple tasks diversity hampers collective performance by reducing the speed of collective problem solving. We find that homogeneous populations are most efficient in optimizing collective payoffs, and diverse populations need more time. This (qualitative) conclusion holds independent of the average degree $\langle k\rangle$. To complement the findings depicted in Fig. 2, in the Supplemental Information (SI) we show results for different types of networks, which show the same qualitative behavior.

### Complex tasks

**Figure 3:**
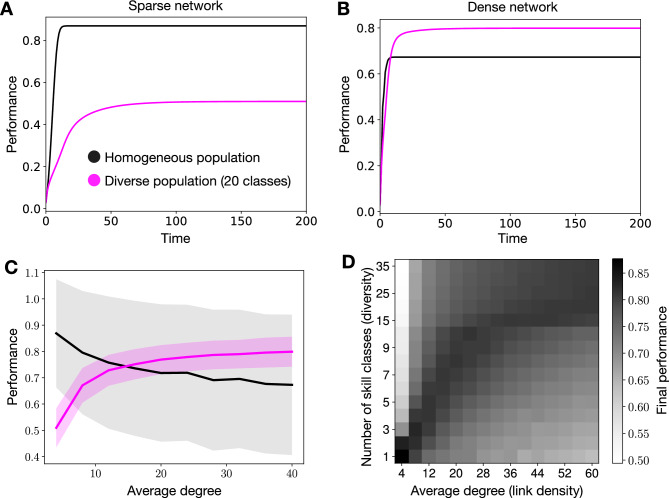
Collective performance in complex tasks. In panels (**A**,**B**), we depict average payoffs over time of homogeneous and diverse populations on sparse ($\langle k\rangle =4$) and more dense ($\langle k\rangle =40$) random networks, respectively. Panel (**C**) depicts the average final payoffs for increasing average degrees of the underlying network. Panel (**D**) shows the average final payoffs (in gray scale) as a function of the average degree of the underlying network and the level of diversity. The reported results are averages over 2500 realizations. The shaded areas in panel (**C**) depict standard deviations. We have tested the statistical significance of the reported performance differences between homogeneous and diverse populations in both sparse and dense networks, see SI.

Figure 3 summarizes the results for complex tasks. In particular, in panels A–C, we compare the collective dynamics and final performance of homogeneous and diverse populations on sparse and dense random networks. In panel D, we systematically vary both the diversity and the average degree of the underlying networks over wider ranges.

In Fig. 3A, we depict the collective performance over time on sparse networks with the same average degree as in panels A–C of Fig. 2, i.e., of $\langle k\rangle = 4$. First, we note that both the homogeneous and diverse populations (20 payoff classes) are not able reach the optimal collective performance $\langle P\rangle =1$. Instead, due to the ruggedness of the complex solution space populations tend to converge to globally suboptimal peaks, which reduces the performance to $\langle P\rangle <1$. As in the case of simple tasks, homogeneous populations converge quickly and diverse populations converge more slowly to the final level of collective performance. More importantly, however, we find that the final performance of diverse populations is worse than the one reached by homogeneous populations. As we will see in the following, this is not always the case and homogeneous populations do not generally outperform diverse ones in complex tasks. Instead, the effect of diversity on collective performance depends on the link density of the underlying network.

In Fig. 3B, we show results for a random network with the tenfold average degree as compared to panel A, i.e., $\langle k\rangle = 40$. While fixing all other model parameters, we find that the increased link density significantly impacts the collective dynamics and qualitatively reverses the previous result. Although, again, diverse populations converge more slowly, the final collective performance of diverse populations is superior to the one reached by homogeneous populations. These findings suggest that collective problem solving in networked populations exhibits a transition point with respect to link density above which diversity becomes beneficial and boosts performance. The results shown in Fig. 3 depend on the definition of group performance. In the SI, we show that the impact of network structure on collective performance is much smaller if we focus on the best or worst payoff in the group rather than looking at the average payoff.

To visualize this phenomenon more clearly, Fig. 3C depicts the final performance of homogeneous and diverse populations in networks of increasing average degree. For low average degrees, homogeneous populations outperform diverse ones, i.e., final solutions found by homogeneous populations have on average higher payoffs. However, as $\langle k\rangle$ increases, diversity becomes beneficial and the final collective performance reached by diverse populations exceeds the one of homogeneous populations. As we show in the SI, this result holds in the limiting case of a fully connected network, where the final collective performance is lowest for homogeneous populations and improves for higher levels of diversity.

In Fig. 3D, we shed further light on the combined effect of link density and diversity by varying both simultaneously. In gray scale we show the final collective performance as a function of the average degree and the number of skill classes. As we have already learnt from Fig. 3C, the performance of homogeneous populations is impaired as the average degree of the network increases. Similarly, on sparse networks the introduction of diversity, i.e., the increase of the number of skill classes, leads to a strong decrease in performance. This is not the case for networks with a higher link density (increasing values of $\langle k \rangle$). Instead, we find that the more dense the network becomes, the higher is the *ideal* level of diversity, which optimizes the final collective performance of the population. To demonstrate that these findings are not restricted to random networks with Poisson degree distribution, we performed additional simulation on random networks with fixed degree and small world networks, see SI.

## Discussion

While various experimental works have found strong effects of diversity on collective performance, theoretical studies that provide a mechanistic understanding of the underlying dynamics induced by the interaction between diversity and network structure are scarce. Here, we fill this gap by means of a simple model of social learning that we extend by agents that differ with respect to their skills. In line with previous studies, we find that diversity can be both beneficial and detrimental to collective performance^13–24,37^. Our modeling approach allows to draw intuitive conclusions on the effects of diversity that are related to the underlying network structure and the complexity of the task.

For simple problems, all populations—regardless of their level of diversity—finally reach the optimal collective performance. Thus to gauge the effect of diversity in simple tasks, we have quantified how much time it takes for populations to converge to the optimal collective performance, $\langle P\rangle =1$. We find that diversity generally slows down the speed of convergence towards optimal performance and therefore hampers collective problem-solving in terms of efficiency. While this effect is more pronounced on sparse networks, it holds qualitatively true on densely connected networks. In the case of complex tasks, the situation is more differentiated. There is no constant effect of diversity on collective problem solving. Instead, the effects of skill diversity depend on the underlying network structure. On sparse networks with a low average degree, diversity impairs collective problem-solving and diverse populations perform worse as compared to homogeneous ones. However, as soon as networks become sufficiently dense diversity becomes beneficial and can boost collective performance beyond that of homogeneous populations.

There are two separate effects introduced by skill diversity that allow us to qualitatively understand the presented findings. First, in diverse populations a particular solution $S_i$ is not evaluated equally by all agents. Thus, a solution may be profitable for some agents (they can extract a high payoff value from it), but not for others (low payoff): we generally find $P_\alpha (S_i)\ne P_\beta (S_i)$ as depicted in panel B of Fig. 1. This feature of diversity introduces noise to the dynamics of collective problem solving. It negatively affects the collective filtering and efficient dissemination of solutions throughout the population. In the case of complex tasks, there is a second effect of diversity that is connected to the ruggedness of the solution space. Here, it is not only that two agents with different skill distributions extract different payoff *values* from a particular solution $S_i$. Additionally, the local neighborhood of a solution in the payoff space may differ. Indeed, while $S_i$ may correspond to a local peak, or maximum, in the payoff function of one agent where they can get stuck, $S_i$ potentially corresponds to a local minimum for a differently skilled agent. The second agent is then able to improve upon the solution by local exploration. This feature gives rise to a mechanism that helps agents in diverse populations to escape suboptimal solutions to complex task. In other words, precisely because two agents have *different* skill sets (and payoff functions), they can benefit from mutual information sharing in order to improve their solutions.

In simple tasks, the payoff landscape is smooth and agents are able to reach solutions that lead to optimal performance, even by individual and local exploration only^38^. Copying from peers with identical skills accelerates this process as time resources spent on individual learning are saved^31^. In essence, solutions are evaluated equally by all agents and homogeneous populations can efficiently filter and disseminate beneficial solutions. The same process is disrupted in diverse populations, as agents with different skills do not agree on the value of a particular solution ($P_\alpha (S_i)\ne P_\beta (S_i)$), ultimately decreasing the convergence rate towards optimal collective performance. The finding is in line with empirical studies, suggesting that diversity often reduces the ability of groups to communicate efficiently, thereby creating an obstacle for collective problem solving in simple tasks^15^. In complex tasks, the situation is different and diversity can boost collective performance: diverse populations outperform homogeneous ones on densely connected networks. As argued previously, homogeneous populations rapidly collapse to, and get trapped at, suboptimal solutions when they copy solutions from their best performing peers^2,3^. Noise from various sources, originating either from different social learning strategies, less well-connected networks or distrust between individuals, has been shown to lead to better exploration of the solution space and improved performance^2–4,39^. Here we identify diversity as another way to boost collective performance. Different skill sets that map onto distinct payoff functions allow agents to mutually assist each other in escaping local optima, a benefit of diversity that is based on the information exchange among *dissimilar* peers.

While our work provides a novel perspective on the relationship between diversity, network structure, and collective performance it comes with noteworthy limitations. We considered the specific type of diversity, where the population can be subdivided in equally sized skill classes. Generally, however, it can be assumed that groups of any kind are not of the same size. As previously shown for different types of social dynamics ranging from collective opinion change to the emergence of conventions, a broader distribution of group sizes can affect system level outcomes^40,41^. An interesting extension to our presented results could furthermore consider different task complexities simultaneously. More specifically, our framework allows to investigate cases where a particular task is complex for most agents in the population, while others can solve it easier. This may shed light on the role of diversity in the context of generalists and specialists, which has previously been studied for homogeneous populations^29^. Moreover, future work building on our proposed modeling framework could explore how the network position of single—and differently skilled—individuals affects collective dynamics to make problem-solving interventions more efficient.

Overall, our findings suggest that diversity offers a pathway to boost collective performance in complex tasks. Specifically, we have shown that the more we are connected, the more we can benefit from diversity to solve complex problems—a finding which informs the compilation of problem solving teams in an increasingly interconnected and diverse world.

### Supplementary Information

Supplementary Information 1.

## Data Availability

The code to produce the raw data analysed in this paper is available at https://github.com/naibaf2/diversity.

## Code availability

Simulation code is available at https://github.com/naibaf2/diversity.
