# Supplementary material for: Network structure shapes the impact of diversity in collective learning
Source: Sci Rep. 2024 Jan 30;14:2491. doi: 10.1038/s41598-024-52837-3 (PMC10827803; doi:10.1038/s41598-024-52837-3)
Supplement: Supplementary file 1 — Supplementary Information 1. [file 41598_2024_52837_MOESM1_ESM.pdf]

Supplemental Information to *Network Structure shapes the Impact of Diversity in Collective Learning*

Fabian Baumann, Agnieszka Czaplicka, and Iyad Rahwan

Center for Humans and Machines,
Max Planck Institute for Human Development, Lentzeallee 94, Berlin 14195, Germany.

In this Supplemental Information, we report results which demonstrate that our main findings on the effects of skill diversity hold for varied system sizes and different types of networks.

Simple tasks

In Fig. 1, we show results for the collective performance in simple tasks for different types of networks. All other model parameters are identical to those considered in Fig. 2 of the main text.

Panel A shows results for random networks that are generated by the configuration model with a constant degree of $k = 4$, i.e., with a degree distribution $p(k) = \delta(k-4)$. In panel B, we consider the case of small-world networks, generated by the Watts-Strogatz model.

As for random networks with a Poisson degree distribution (considered in the main text), we find that in both cases, i.e., for random networks with fixed degree and small-world networks, diversity is detrimental in simple tasks: the time to reach optimal performance increases with diversity.

Complex tasks

Performance on a fully connected network

Figure 4 shows the collective performance over time obtained on a fully connected network for a complex task. Different colors correspond to different levels of diversity.

In the main text, we have reported that in complex tasks the benefits of diversity increase for increasing link densities of the underlying random networks. Here we show that the result holds in the limit of a fully connected network: final collective performance increases with increasing levels of (skill) diversity.

Varying the system size

In the main text, we considered populations of size $N = 1000$. In Fig. 2, we show additional results for different system sizes.

In particular, we reproduced Fig. 3 (panels A and B) of the main text with half ($N = 500$, Fig. 2A) and double the system size ($N = 2000$, Fig. 2B). As expected, we find that the final values of the collective performance differ slightly. In general, higher values of N lead to better performance, due to the fact that large populations can sample the payoff landscape more thoroughly than small populations.

More importantly, however, the qualitative behavior reported in the main text is recovered. In both cases, for smaller (panel A) and larger (panel B) system sizes, diverse populations outperform homogeneous ones on dense networks, while the opposite is true for sparse networks.

Different network models

In Fig. 3, we reproduced Fig. 3 (panels A and B) of the main text for different types of networks, while fixing all other model parameters.

In panel A, we show results for random networks generated by the configuration model with fixed degree k, which are either sparse ($k = 4$, left) or dense ($k = 40$, right). In panel B, we consider the case of sparse ($\langle k \rangle = 4$) and dense ($\langle k \rangle = 40$) small-world networks.

In both cases our main conclusion holds: link density modifies the effect of diversity, and diverse populations outperform homogeneous ones in densely connected networks.

Different performance measures

In Fig. 5, we reproduced Fig. 3 (panels A and B) of the main text for different types different performance measures, while fixing all other model parameters. In particular, we consider the best (BP) and the worst payoff (WP) within the population. While diverse populations perform

better with respect BP, homogeneous populations perform better when considering WP. This qualitative picture is robust with respect to changing the link density of the social network (from low to high).

Statistical tests

To make our analysis more robust, we conducted statistical tests on the key findings presented in Fig. 3 of the main text. Specifically, we utilized both two-sample Kolmogorov-Smirnov tests and T-tests to assess the significance of the observed differences in final average performance between homogeneous and diverse populations in networks with sparse connectivity (average degree $\langle k \rangle = 4$) and dense connectivity (average degree $\langle k \rangle = 40$). Given the number of simulations (2500 realizations per condition), we obtained extremely small p-values for both types of tests and across both network conditions. Specifically, we found p-values of 0.0 for both the KS-test and T-test in the case of sparse networks ($\langle k \rangle = 4$), and p-values of 2.5^{-250} for the KS-test and 7.3^{-112} for the T-test in the case of dense networks ($\langle k \rangle = 40$). Consequently, we can confidently conclude that our results regarding the performance difference between homogeneous and diverse populations hold strong statistical significance.

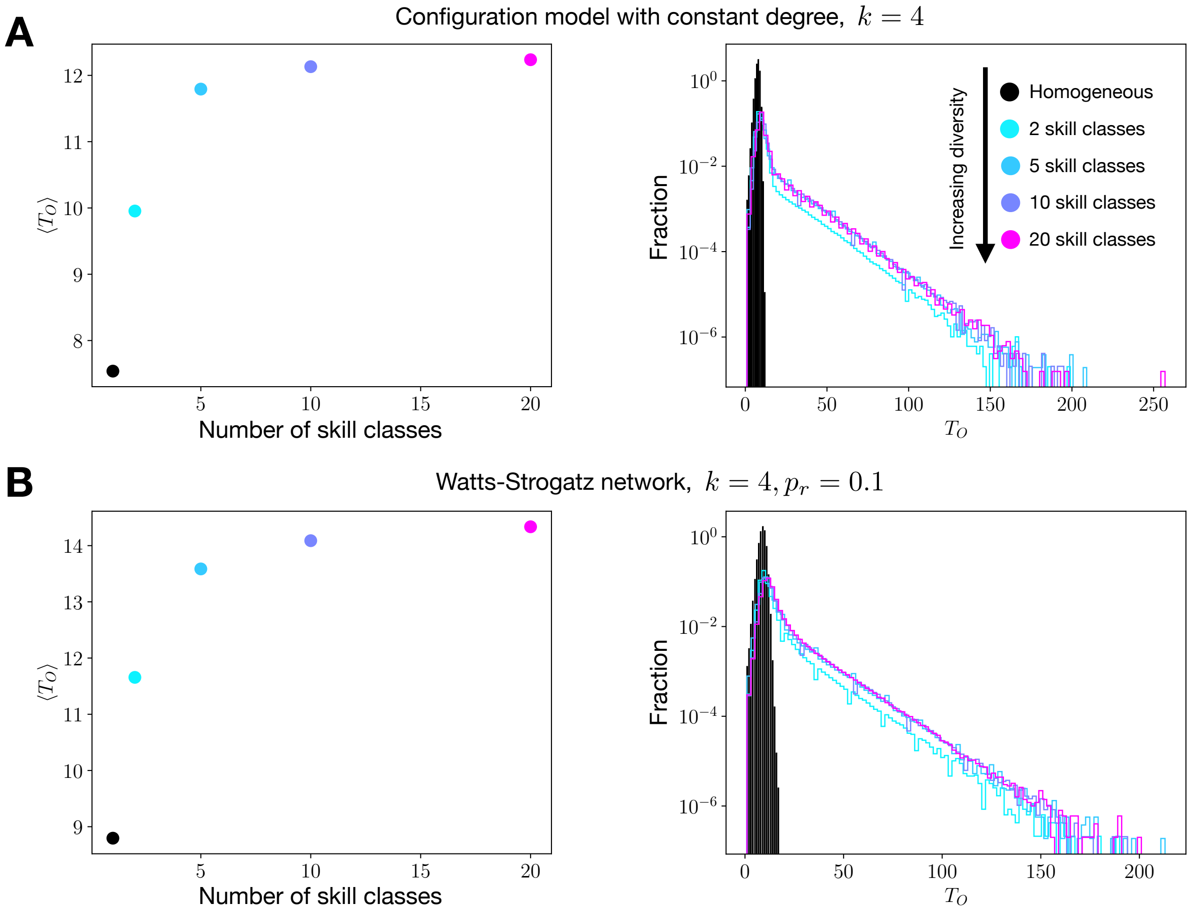

Figure 1: Collective performance in simple tasks. Mean times $\langle T_0 \rangle$ to reach optimal performance (left) and the corresponding distributions of T_0 for different levels of diversity (right). In panel A, we show results for random networks generated by the configuration model with a fixed degree of $k = 4$, i.e. each node has four adjacent nodes. In panel B, we show results for small world networks generated by the Watts-Strogatz model, where each link of a regular grid network is rewired with probability p_r. Here we chose a regular gird network where each node is connected to its $k = 4$ nearest neighbors and set the rewiring probability to $p_r = 0.1$. In all shown cases the system size is $N = 1000$. The results are averaged over 2500 realizations.

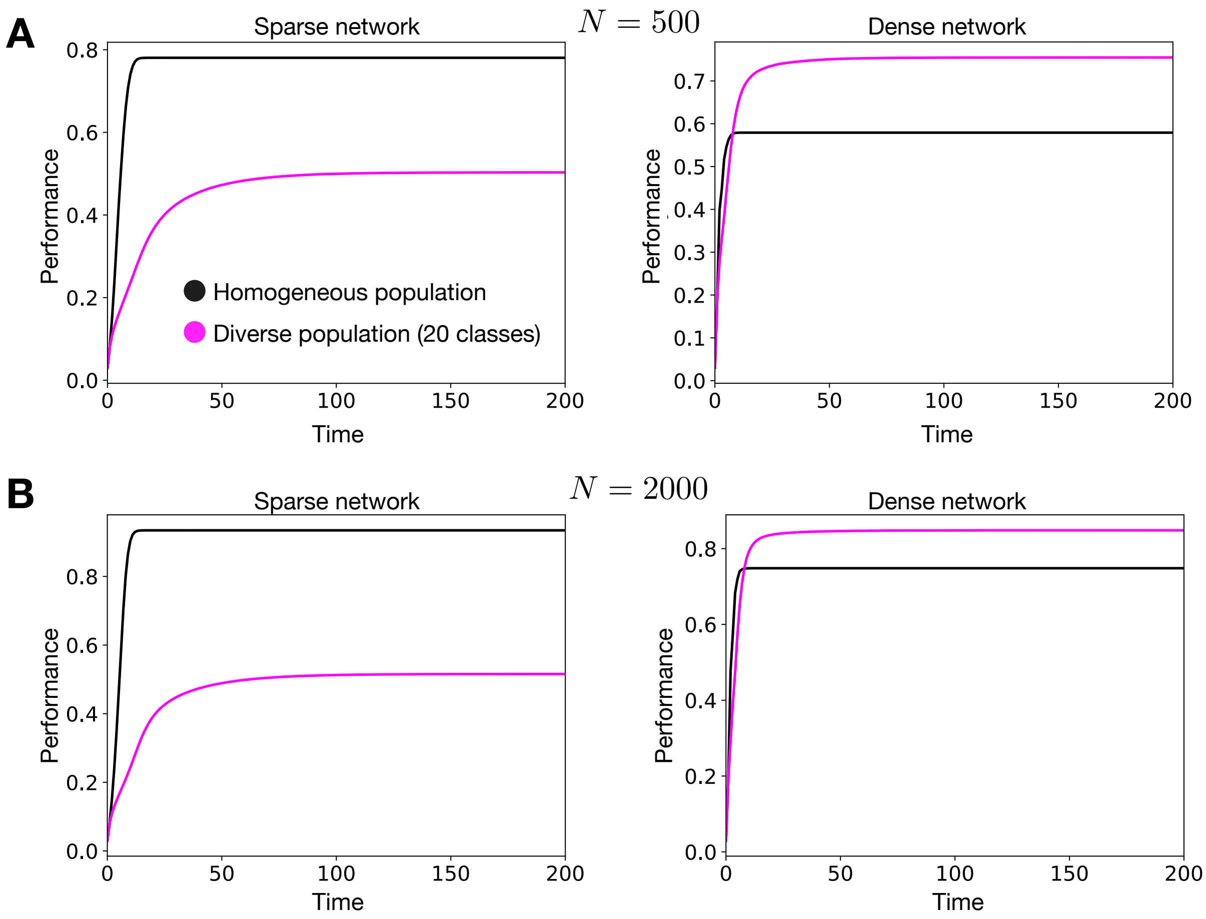

Figure 2: Collective performance in complex tasks for $N = 500$ (panel A) and $N = 2000$ (panel B). The average degrees of the underlying random networks are identical to those of Fig. 3 (panels A and B) of the main text, i.e., we set $\langle k \rangle = 4$ and $\langle k \rangle = 40$ for sparse and dense networks, respectively. The results are averaged over 2500 realizations.

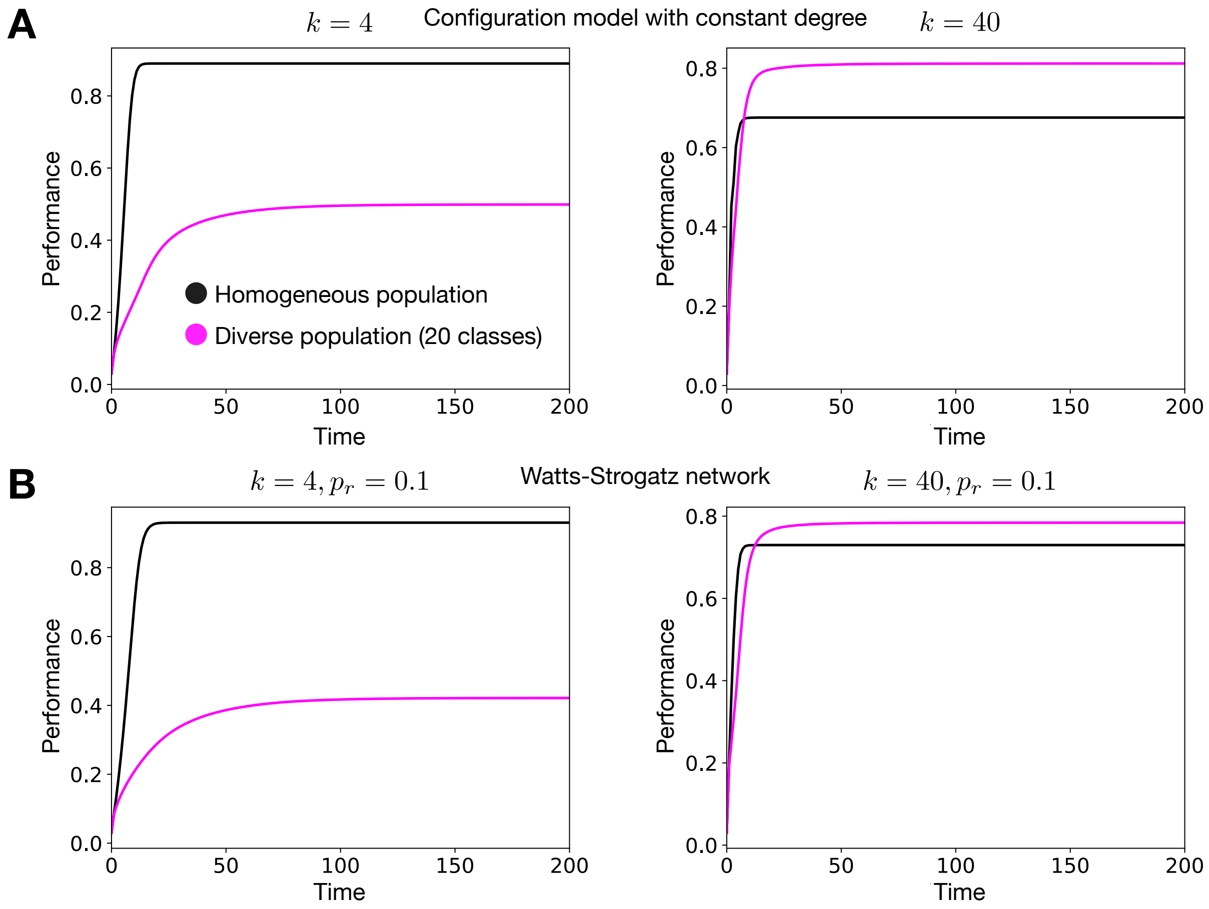

Figure 3: Collective performance in complex tasks. Panel A shows results for networks generated by the configuration model with a constant degree for each node: $k = 4$ (left) and $k = 40$ (right). In panel B, we show results for small-world networks generated by the Watts-Strogatz model, where each link of a regular grid network is rewired with probability p_r. Here we chose a regular gird network where each node is connected to its $k = 4$ (left) and $k = 40$ (right) nearest neighbors and set the rewiring probability to $p_r = 0.1$. In all shown cases the system size is $N = 1000$. The results are averaged over 2500 realizations.

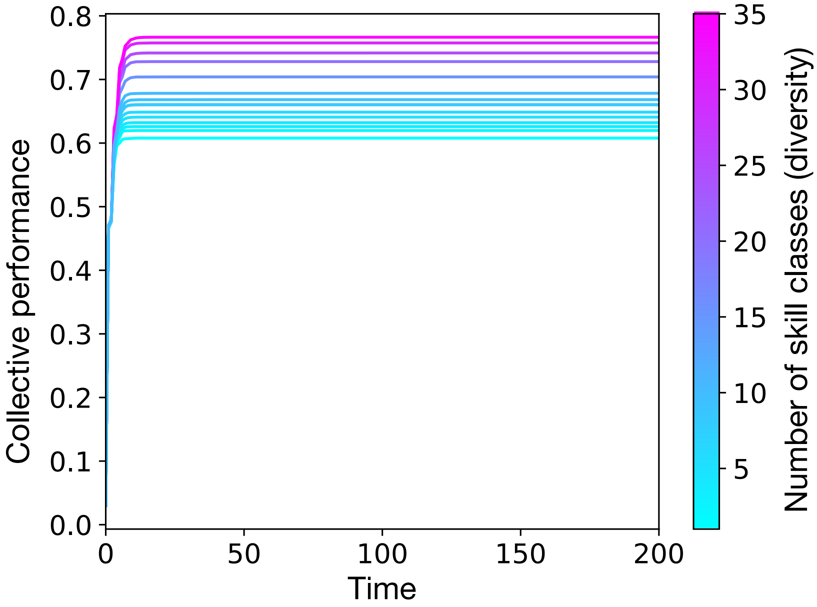

Figure 4: Collective performance over time for populations with increasing levels of diversity (number of skill classes) on a fully connected network. The colors of the trajectories encode the number of skill classes in populations of $N = 1000$ agents. The results are averaged over 2500 realizations.

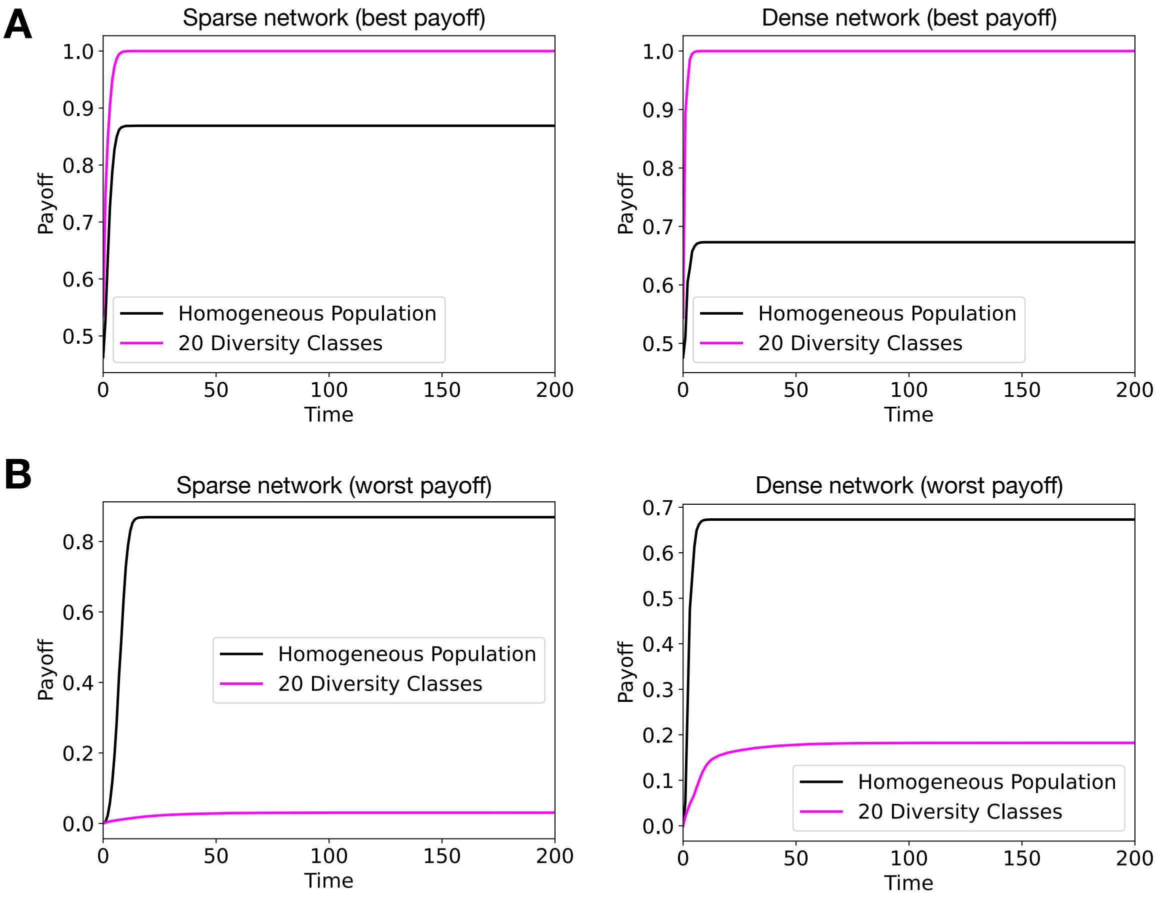

Figure 5: Dynamics over time for complex tasks and different measures of collective performance. Panel A and B show the results for using the best and the worst payoff in a population as a measure for collective performance, respectively. All model parameters are identical to those of Fig. 3 (panels A and B) of the main text. The results are averaged over 2500 realizations.
